# Meta-Analysis of Isolated Hepatic Perfusion and Percutaneous Hepatic Perfusion as a Treatment for Uveal Melanoma Liver Metastases

**DOI:** 10.3390/cancers13184726

**Published:** 2021-09-21

**Authors:** Martijn S. Bethlehem, Dimitrios Katsarelias, Roger Olofsson Bagge

**Affiliations:** 1 Department of Surgery, Sahlgrenska University Hospital, 413 45 Gothenburg, Sweden; dimitrios.katsarelias@gu.se (D.K.); roger.olofsson.bagge@gu.se (R.O.B.); 2 Institute of Clinical Sciences/Sahlgrenska Academy, University of Gothenburg, 413 90 Gothenburg, Sweden; 3 Wallenberg Centre for Molecular and Translational Medicine, University of Gothenburg, 413 45 Gothenburg, Sweden

**Keywords:** uveal melanoma, liver metastases, isolated hepatic perfusion, percutaneous hepatic perfusion

## Abstract

**Simple Summary:**

Isolated hepatic perfusion is one of the available treatment options for patients with liver metastases from uveal melanoma. This is an open surgical procedure where the liver is isolated from the circulation and perfused with a chemotherapeutic agent. A modern development is the minimally invasive percutaneous hepatic perfusion, where the liver is endovascularly isolated and then perfused with a chemotherapeutic agent through a catheter in the arterial system. Within this systematic review and meta-analysis, we aim to compare these modalities in terms of overall survival, progression-free survival, complications and response.

**Abstract:**

Background: Uveal melanoma is the most commonly occurring primary intraocular malignancy in adults, and patients have a high risk of developing metastatic disease, mostly in the liver. Isolated hepatic perfusion (IHP) with melphalan is a liver-directed therapy for patients with liver metastases. Percutaneous hepatic perfusion (PHP), a minimally invasive technique, is available as well. PHP benefits from the fact that the procedure can be repeated and therefore possibly offers better survival. We conducted a systematic review and meta-analysis comparing both techniques. Methods: A systematic literature search was performed using the electronic databases of Scopus, MEDLINE, Web of Science, PubMed and Cochrane CENTRAL. A total of nine articles reporting on eight studies were included in the analysis. Individual survival data were extracted from each study. Results: The median overall survival (OS) was 17.1 months for IHP and 17.3 months for PHP. The median progression-free survival (PFS) was 7.2 months for IHP and 9.6 months for PHP. The median hepatic progression-free survival was 10 months for IHP and 9.5 months for PHP. The complication rate and 30-day mortality rate were 39.1% and 5.5% for IHP and 23.8% and 1.8% for PHP. Conclusion: There was no difference in OS or PFS between IHP and PHP for patients with uveal melanoma liver metastases, but patients have significantly less of a risk for complications and mortality following PHP.

## 1. Introduction

Uveal melanoma is the most commonly occurring primary intraocular malignancy in adults [1]. It is very different from cutaneous melanoma, considering clinical behavior, response to treatment and known mutations [2,3]. In the United States, uveal melanoma accounts for approximately 5% of all new melanoma patients [2,4,5]. In 80–85% of the patients with uveal melanoma, the tumor arises from melanocytes in the choroid region, while the remaining tumors arise from the iris and ciliary body [1,6,7]. Uveal melanoma has a poor prognosis, as 25–31% of the patients will develop metastases within 5 years, 34–45% within 10–15 years and 49% within 25 years [8,9]. When looking at subgroups of baseline tumor dimensions (longest basal diameter and apical height), patients with smaller tumor dimensions have a lower risk of metastasizing than those with increased dimensions [9]. The liver is the most common site for metastases, and it is involved in up to 90% of cases for patients with metastatic disease. Other common metastatic sites are the lungs and skeleton [1,7,9,10,11,12].

Liver-directed therapies are being used in an attempt to cure or stabilize liver metastases in patients with the disease. This meta-analysis focuses on hepatic perfusion, but other liver-directed therapies include liver surgery, intra-arterial therapies (e.g., hepatic artery infusion/HAI, trans-arterial chemoembolization/TACE, selective internal radiotherapy/SIRT, immunoembolization/IE) and hyperthermic therapies (e.g., radio frequent ablation/RFA and laser-induced interstitial thermotherapy/LITT). Systematic reviews and meta-analyses of liver-directed therapies other than hepatic perfusion have shown that they do not provide a significantly higher survival rate as a benefit when compared to the best alternative care [13,14,15,16,17].

Isolated hepatic perfusion (IHP) was first applied 60 years ago to treat liver metastases [18]. First, the vena cava circulation is bypassed through a veno-venous shunt from the femoral vein to the external jugular vein. Then, using laparotomy, the liver is isolated from the systemic circulation. This involves extensive dissection, after which a catheter is positioned in the proper hepatic artery, while another catheter is positioned in the infrahepatic caval vein. The catheters are connected to a heart–lung machine and the liver is perfused with a high dose of chemotherapy under hyperthermia [19].

The first reported use of the minimally invasive technique to perform a percutaneous hepatic perfusion (PHP) was in 1994 [20]. In this procedure, a catheter is placed percutaneously in the proper hepatic artery for the infusion of chemotherapy. A second, double-balloon catheter is placed in the inferior caval vein to prevent leakage to the systemic circulation. The catheter is fenestrated between these balloons to aspirate the blood coming from the hepatic veins, that is then run through an extra-corporeal filtration system before returning to the patient through a third catheter in the jugular vein [21]. This minimally invasive technique was developed to reduce the morbidity and mortality related to the open procedure and to reduce the length of the procedure. Another benefit is that the percutaneous procedure can be repeated, which could potentially have a positive effect on response and survival.

Today, both IHP and PHP are used for the treatment of liver metastases from uveal melanoma, but a comparison of the two methods has never been reported in the literature. The aim of this study was to perform a systematic review and meta-analysis comparing these techniques in terms of overall survival, progression-free survival, response and complications.

## 2. Materials and Methods

This study was conducted in accordance with the Meta-analysis of Observational Studies (MOOSE) reporting guidelines [22]. The study protocol was designed prospectively and registered online with PROSPERO, the international prospective register of systematic reviews (PROSPERO ID CRD42021255132) [23].

### 2.1. Database Search Methodology

A systematic literature search was conducted across the Scopus, Ovid MEDLINE, PubMed, Web of Science and Cochrane CENTRAL electronic databases. The primary outcomes were overall and progression-free survival, with the risks of complications, mortality and response as secondary outcomes.

The search strategy carried out in the Scopus database was as follows: (uveal melanoma OR choroidal melanoma OR ciliary body melanoma OR ciliochoroidal melanoma OR iridociliary melanoma OR iris melanoma OR intraocular melanoma OR ocular melanoma) AND (metast *) AND (perfusion). For Ovid MEDLINE: (uveal melanoma OR choroidal melanoma OR ciliary body melanoma OR ciliochoroidal melanoma OR iridociliary melanoma OR iris melanoma OR intraocular melanoma OR ocular melanoma) AND (perfusion). For PubMed and Web of Science: (uveal melanoma OR choroidal melanoma OR ciliary body melanoma OR ciliochoroidal melanoma OR iridociliary melanoma OR iris melanoma OR intraocular melanoma OR ocular melanoma) AND (metast * OR stage IV) AND (perfusion). Additionally, for Cochrane CENTRAL: “(uveal neoplasms):ti,ab,kw AND (melanoma):ti,ab,kw AND (neoplasm metastasis):ti,ab,kw” (word variations have been searched). The database search was conducted on 26 May 2021.

### 2.2. Inclusion Criteria

The inclusion criteria were: original research studies on hepatic perfusion for uveal melanoma metastases written in English, containing search text words, basic patient characteristics and a table or Kaplan–Meier curve for survival data. Because of the evolution of liver perfusion techniques, the publication search range was set between 1 January 2000 to the present date (26 May 2021). Duplicate records were excluded. Two authors (M.S.B., R.O.B.) reviewed the remaining titles to confirm that the subject was the treatment of uveal melanoma metastases in the liver with either IHP or PHP. Review articles were excluded. Articles on animal models, laboratory investigations, imaging, primary or locally recurrent tumors, prognosis, staging and quality of life were excluded. Articles that included primary cutaneous or mucosal melanoma were excluded unless patients with uveal melanoma were reported separately. If it was uncertain whether patients in any articles from the same study group overlapped, we excluded the article(s) with fewer patients. Finally, we compared reference lists with our search and archives to identify additional articles.

### 2.3. Data Extraction

Data were extracted from the included articles and transferred to a standard sheet including the title, first author, year of publication, characteristics of study design and study participants as well as overall survival (OS), hepatic progression-free survival (hPFS), overall progression-free survival (PFS), response and reported morbidity and mortality. The survival times were defined as the time elapsed from treatment with either IHP or PHP to censorship or the event of interest: death for OS, tumor progression in the liver for hPFS and tumor progression in the whole body for PFS. Responses were compared between articles that reported responses according to the Response Evaluation Criteria in Solid Tumors (RECIST) and revised RECIST guideline (RECIST 1.1) [24,25]. Morbidity was compared considering Clavien–Dindo grade III or IV complications [26] (not including hematotoxic complications related to the chemotherapeutic agent used) and mortality was compared using reported 30-day mortality rates.

A database combining all available individual patient data from the different articles on OS, hPFS and PFS was created. If there were no numerical individual patient data presented in the text or a table, we used WebPlotDigitizer version 4.4 (a web-based plot digitizing tool available to users free of charge) to extract survival data from the available Kaplan–Meier plots or other graphs [27]. After launching the digitizer, users upload a screenshot of an XY chart and are then prompted to calibrate the axes. After calibrating, the user manually clicks each data point within the data series and then downloads the extracted coordinates as a spreadsheet. Studies have shown high levels of intercoder reliability and validity [28,29]. By using this application, survival times for each step for deaths, and a tick for each censored event, were obtained. Where censored events were not displayed, but a numbers-at-risk table was available, the at-risk reduction minus deaths was taken to be the number of censored events during each interval, which was then assigned to its midpoint [30]. Finally, a new Kaplan–Meier curve was created with the extracted data using SPSS, Version 27.0.1.0 (IBM SPSS Statistics for Macintosh, Armonk, NY, USA: IBM Corp.), then copied to Affinity Photo, version 1.9.3 (Serif (Europe) Ltd., Nottingham, UK), and layered with the original graph to verify that a matching curve was obtained.

### 2.4. Statistical Analysis

Kaplan–Meier estimations were used to assess OS, hPFS and overall PFS. OS data were censored if patients were stated as alive in descriptive text or table. The log-rank test was used to compare curves. For descriptive statistics, a Chi-square test was used to compare outcomes. *p* values of <0.05 were considered statistically significant. Analyses were performed using SPSS, Version 27.0.1.0 (IBM SPSS Statistics for Macintosh, Armonk, NY, USA: IBM Corp.).

## 3. Results

### 3.1. Study and Patient Characteristics

**Figure 1:**
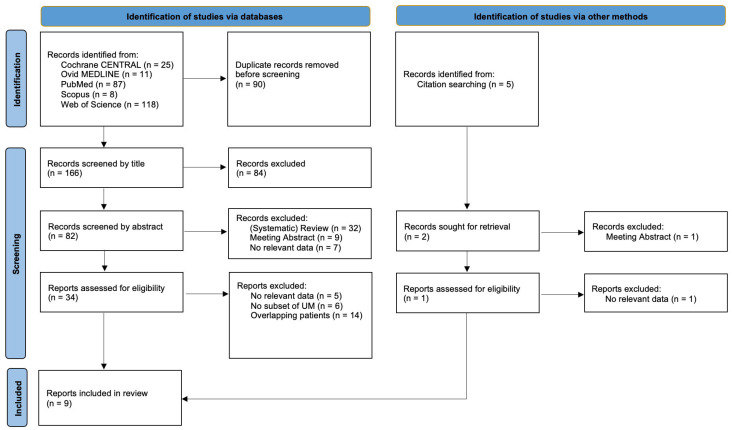
Search flow diagram. UM: uveal melanoma.

The search strategies generated 256 identified records (Scopus *n* = 15; Ovid MEDLINE *n* = 11; PubMed *n* = 87; Web of Science *n* = 118; Cochrane CENTRAL *n* = 25), where 90 records were excluded due to duplications. The remaining 166 titles were screened, which led to the exclusion of another 84 records. The abstracts of the remaining 82 articles were extracted and screened and another 48 articles were excluded. A citation search of the remaining articles was performed, and this identified five additional records. Of these, three were excluded based on their title. After screening the other two abstracts, only one additional full-text article was included. Finally, all 35 selected full-text articles were screened and assessed for eligibility. Five articles were excluded due to a lack of relevant data [31,32,33,34,35], seven articles were excluded due to not reporting a separate subset of patients with liver metastases of uveal melanoma [36,37,38,39,40,41,42] and fourteen articles were excluded due to an overlap in the reporting of the included patient series with other articles from the same study groups [43,44,45,46,47,48,49,50,51,52,53,54,55,56]. A more detailed description of the decisions made in the exclusion process is provided in Supplement file 1. In total, nine articles describing eight different patient series were included in the systematic review (Figure 1).

The study design differed between the eight reported patient series: one combined reporting of a phase I + phase II clinical trial, one phase II clinical trial and six retrospective cohorts. There were six single-center reports and two multicenter studies. The mean number of participants in the included studies was 37 (range 19–68). Patients were treated with IHP in three and with PHP in five of the reported series (Table 1). In total, 292 patients were included in the meta-analysis, of which 128 patients underwent IHP and 164 patients PHP. The total number of PHP procedures was 367 with a median of two procedures per patient (range 1–6). Further baseline characteristics of the patients in the eight included series are provided in Table 2.

### 3.2. Overall Survival

**Figure 2:**
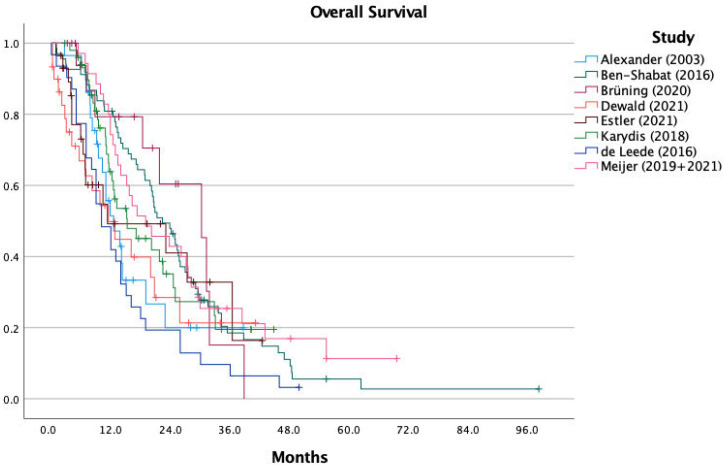
Overall survival per study.

**Figure 3:**
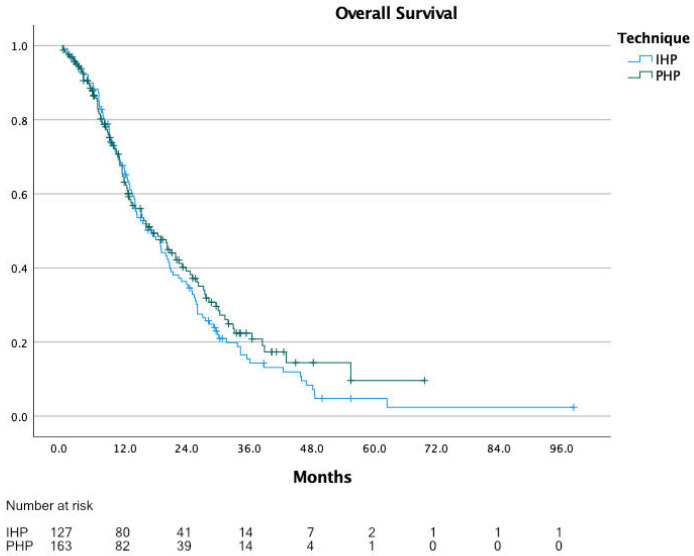
Overall survival comparing IHP vs. PHP.

The range of the reported median OS in the eight included studies was 10.0 to 22.4 months, with a calculated median OS of 14.1 months for all patients combined. For IHP, the median OS was 12.1 months (range 10.0 to 22.4 months) and for PHP, 15.3 months (range 12.0 to 19.1 months). Figure 2 shows the KM curves of all included studies combined in one graph. For OS, individual patient data were extracted for all 292 patients, with a calculated median OS of 17.1 months for all patients, 17.1 months for IHP and 17.3 months for PHP (*p* = 0.366) (Figure 3).

### 3.3. Progression-Free Survival

**Figure 4:**
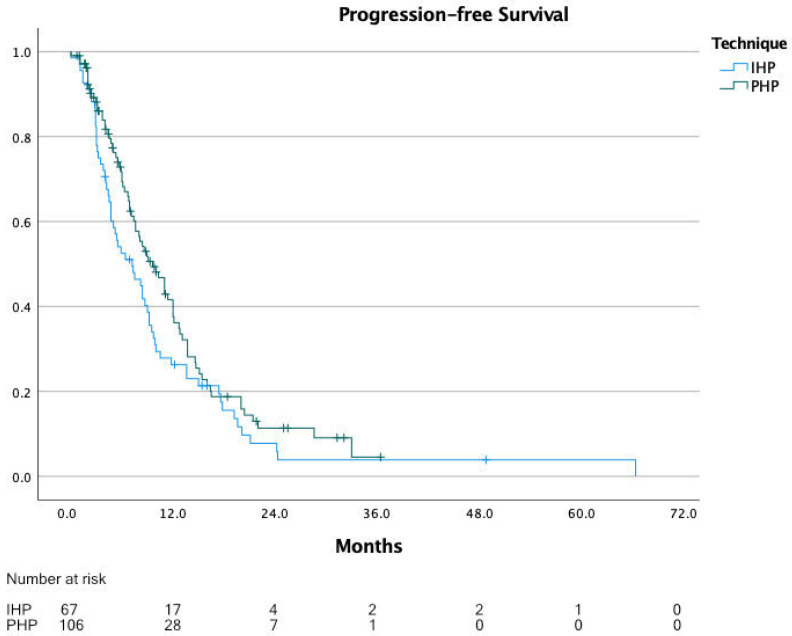
Progression-free survival.

The range of the reported median PFS was 6.0 to 14.3 months, with a calculated median PFS of 7.8 months for IHP and PHP patients combined. For IHP, the median PFS was 8.0 months (range 6.0 to 13.9 months) and for PHP, 7.6 months (range 6.0 to 14.3 months). For PFS, individual patient data were extracted for 175 patients, of which 68 underwent IHP and 107 underwent PHP. The calculated median PFS from these data was 8.4 months for all patients, 7.2 months for IHP and 9.6 months for PHP (*p* = 0.094) (Figure 4).

### 3.4. Hepatic Progression-Free Survival

**Figure 5:**
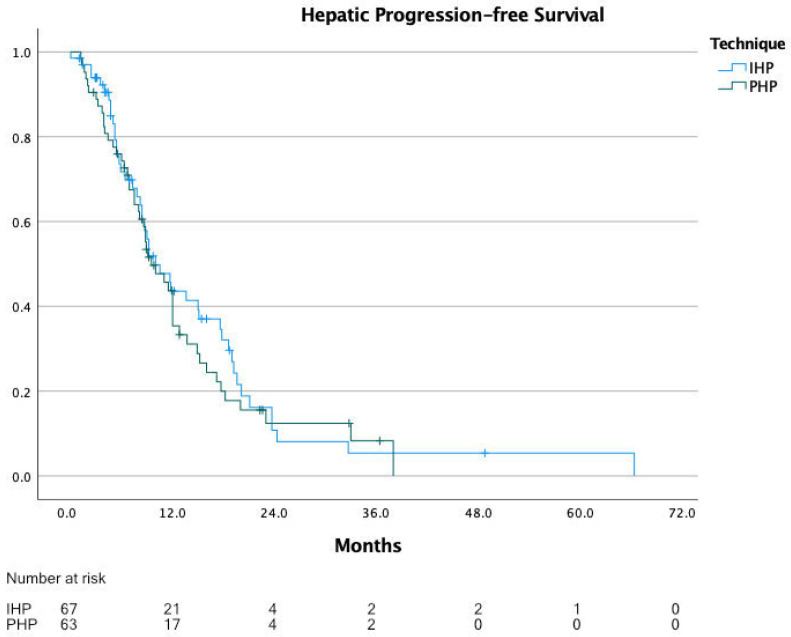
Hepatic progression-free survival.

The range of reported median hPFS was 6.0 to 12.0 months, with a calculated median progression-free survival of 10.0 months for IHP and PHP patients combined. It is to be noted that three studies did not report hPFS. For IHP, the median hPFS was 11.0 months (range 10.0 to 12.0 months), and for PHP, 9.1 months (range 6.0 to 11.2 months). For hPFS, individual patient data were extracted for 131 patients, of which 68 underwent IHP and 63 underwent PHP. The calculated median hPFS from these data was 10.0 months for all patients, 10.0 months for IHP and 9.5 months for PHP (*p* = 0.544) (Figure 5).

### 3.5. Complications and Mortality

The three studies on IHP reported a total of 50 Clavien–Dindo Grade III or IV complications in a total of 128 patients (39.1%) and the five studies on PHP reported a total of 39 complications in a total of 164 patients (23.8%) (*p* = 0.005). The reported 30-day mortality for IHP was seven patients (5.5%) and for PHP, three patients (1.8%) (*p* = 0.09). When calculated in relation to the number of performed PHP procedures (367 in total), and compared to IHP, the Clavien–Dindo Grade III or IV complication rate was 10.8% (*p* < 0.001) and the 30-day mortality rate was 0.8% (*p* = 0.001).

### 3.6. Response

Two studies on IHP reported response rates, including 15 complete responses (15.5%, *p* < 0.001) and 44 partial responses (45.4%) in a total of 97 patients. The five studies on PHP reported 4 complete responses (2.4%) and 76 partial responses (46.3%) in a total of 164 patients.

## 4. Discussion

The calculated OS data show that there is no difference between IHP and PHP for patients with liver metastases from uveal melanoma in the long term, but that patients have a significantly lower risk of complications and mortality following PHP.

All three studies reporting on IHP excluded patients with extra-hepatic disease, whereas four out of the five studies on PHP included patients with extra-hepatic disease if it was either “limited” or the patients had a liver-dominant metastasizing pattern. The study that included most patients with extra-hepatic disease (12 out of 29 patients) found no difference in OS between patients with or without these extra-hepatic metastases at the time of PHP [61]. One study on IHP excluded patients with a tumor burden of more than 50% of the liver volume [63]. In another study, these patients were excluded in the last of three periods, because tumor burden >50% was associated with higher mortality for the patients included in the first two periods [58]. For PHP, only one study mentions a maximum tumor burden of 60% [64,65]. Of all patients included in the IHP studies, the majority did not receive any prior treatment for their liver metastases. In at least two out of the five articles on PHP, most of the patients had prior treatment for their liver metastases. All the previously mentioned exclusion criteria, or characteristics of the included patients, could imply a selection bias from which survival rates after IHP could benefit.

Overall survival data could be extracted for all reported patients in the eight included studies. The difference in outcome, when comparing the results of the reported median survival data or from the combined extracted data, shows that the extraction process is of value. Longer OS for PHP was to be expected as repeated procedures are possible and were performed in most patients, but this was not shown by the data. It might be that the normothermic perfusion during PHP is less effective than the hyperthermic perfusion during IHP [66]. This hypothesis can be supported by the fact that significantly more patients have a complete response after IHP, although this does not lead to a significantly longer hPFS for IHP. The latter may be caused by higher mortality and complication rates after IHP, which may have also limited the duration of response. Survival might also have been influenced by the available systemic treatments over the included years, as the patients treated with IHP were included between 1989 and 2013 and those treated with PHP between 2008 and2020.

PHP proves to be safer than IHP in this meta-analysis. The higher mortality in the IHP group is possibly partially due to the period in which the patients were included. The study on IHP that reports the highest mortality rate (5/68 patients) used different chemotherapeutic strategies, higher temperatures, and included patients with tumor burden over 50% in the earlier years of their cohort. TNF-alpha was not used after the first period in this study due to higher mortality. The dose of melphalan was lowered after the second period in this study, in combination with excluding patients with tumor burden over 50%, due to higher mortality [37,58]. After these changes, the mortality rate was only 1/59 patients (2.5%) [58], comparable to the 1.8% mortality (3/164 patients) reported for PHP. Higher occurrence of Clavien–Dindo grade III and IV complications after IHP can probably be related to major abdominal surgery and to the inclusion of patients with a high tumor burden as well. These complications might even have an influence on the duration of response and/or survival.

To evaluate response, all studies reporting on PHP used the RECIST 1.1 guidelines. In summary, this means that partial response (PR) was defined as a decrease in volume of 30% or more from baseline measurements, and progressive disease (PD) as an increase in volume of 20% or more or one or more new lesions. All the studies reporting on IHP used different definitions. Ben-Shabat et al. used a decrease in volume of 30% or more for PR as well, but used any increase in volume or new lesions as the definition for PD [58]. Alexander et al. used a decrease in volume of 50% or more for PR and an increase in volume of 25% or more as definition for PD [57]. De Leede et al. provide no definition for PR and used an increase in volume of 25% or more as a definition for PD [63]. These differences in definitions could possibly have led to lower partial response rates and longer hepatic progression-free survival times in the IHP group.

Differences in the format of the results presented and methodologies used made it challenging to pool the data from the original publications. For example, some articles did not report definitions on survival times. Others did not supply censored events or at-risk tables along with Kaplan-–Meier curves, so we used estimations for the unreported data to recreate the curves. After combining multiple publications, which increased the number of deaths and censored events, and by adding the extra step of checking that the original and recreated curves overlap, the error margin of the estimated data probably falls within a couple of weeks of the actual survival. Regarding conflicts of interest or possible publication bias, two studies were fully or partially funded by industry, with authors reporting a conflict of interest, or both. One study was externally funded but not by the industry. In four studies, neither the industry nor any external funding was involved and for one study, such information was not available.

After the approval of checkpoint inhibitors for metastasized uveal melanoma, patients more recently diagnosed with progression could probably have a prolonged survival which is not due to their liver-directed treatment. Immunotherapy with nivolumab, pembrolizumab and/or ipilimumab has shown to only have a limited effect for patients with uveal melanoma [13,14,15,16,67,68,69,70,71]. A recent phase II trial combining epigenetic therapy using the HDAC inhibitor entinostat with the PD-1 inhibitor pembrolizumab resulted in durable responses in a subset of patients [72]. Even more promising results were obtained in a phase III study using tebentafusp, a bispecific fusion protein linking melanoma cells with T-cells. This is the first phase III trial to show a significant improvement in OS (22 months vs. 16 months); however, the treatment is only available for patients with an HLA-A*02 serotype, and the increase in survival is still modest compared to the effects of immunotherapy in cutaneous melanoma [73].

The last question, that this meta-analysis cannot answer, is whether hepatic perfusion in any form gives patients a survival benefit over the “best alternative care”. There are currently two ongoing randomized controlled trials on this topic. The SCANDIUM trial is a phase III randomized controlled multicenter trial evaluating whether IHP increases overall survival in comparison with the best alternative care in patients with isolated liver metastases from uveal melanoma [74]. The inclusion for the SCANDIUM trial has just been completed. The FOCUS trial is a phase III randomized controlled multicenter trial comparing PHP with the best alternative care in patients with liver-dominant metastases from uveal melanoma [75]. The preliminary results from the FOCUS trial were recently presented at the 2021 ASCO Annual Meeting, showing a progression-free survival of 9.03 months following PHP compared to 3.06 months with the best alternative care [76]. We eagerly await the survival outcomes of these two trials.

## 5. Conclusions

Overall survival data show that there is no difference between IHP and PHP for patients with liver metastases from uveal melanoma in the long term, but patients could potentially have a lower risk of complications and mortality following PHP.

## Figures and Tables

**Table 1:** Patient series characteristics.

1st Author	Year of Publication	Study Design	No. of Centers (Country)	Number of Patients	Technique	Years of Inclusion
Alexander [57]	2003	Phase I + II	1 (USA)	29	IHP	1997–2002
Ben-Shabat [58]	2016	Retrospective	1 (SWE)	68	IHP	1989–2013
Brüning [59]	2020	Retrospective	1 (DEU)	19	PHP	2014–2019
Dewald [60]	2021	Retrospective	1 (DEU)	30	PHP	2014–2019
Estler [61]	2021	Retrospective	1 (DEU)	29	PHP	2015–2020
Karydis [62]	2018	Retrospective	2 (GBR and USA)	51	PHP	2008–2016
de Leede [63]	2016	Retrospective	2 (NLD)	31	IHP	1999–2009
Meijer [64,65]	2019 + 2021	Phase II	1 (NLD)	35	PHP	2014–2017

USA: United States of America, SWE: Sweden, DEU: Germany, GBR: United Kingdom, NLD: the Netherlands.

**Table 2:** Patient characteristics.

Study	Age (Years) (Range)	Female: Male (No. of Patients)	Tumor Load (No. of Patients)		Extra-Hepatic Disease (No. of Patients, %)
Alexander [57]	49 (26–73)	15:14	<25% 25–50% >50%	20 8 1	Not Included
Ben-Shabat [58]	61 (18–77)	40:28	1–4 met 5–10 met 11–100 met >100 met	11 24 13 4	Not Included
Brüning [59]	58 (range not specified)	8:11	Not specified		Included Number of patients not specified
Dewald [60]	57 (52–66)	21:9	≤30% >30%	21 8	5 (16.7%)
Estler [61]	69.7 (30–81)	18:11	<25% >25%	22 7	12 (41.4%)
Karydis [62]	57.9 (27.9–77.1)	28:23	1–3 met 4–10 met >10 met	12 23 16	8 (15.7%)
de Leede [63]	57 (27–68)	19:12	<50%	31	Not Included
Meijer [64,65]	59 (42–71)	19:16	1–5 met 6–9 met ≥10 met	9 8 18	Not Included

Met: metastases.

## Supplementary Materials

The following are available online at www.mdpi.com/article/10.3390/cancers13184726/s1, Supplement file 1: full-text article selection process.
