# Supplementary material for: Meta-Analysis of Isolated Hepatic Perfusion and Percutaneous Hepatic Perfusion as a Treatment for Uveal Melanoma Liver Metastases
Source: Cancers (Basel). 2021 Sep 21;13(18):4726. doi: 10.3390/cancers13184726 (PMC8469397; doi:10.3390/cancers13184726)
Supplement: Supplementary file 1 [file cancers-13-04726-s001.zip › cancers-1377385-supplementary.pdf]

Supplement 1 - full-text article selection process

As mentioned in the results section of the main text, 35 articles were screened and assessed for eligibility. In this process five articles without relevant data were excluded first; Buzzaco *et al* because of high selection bias and not reporting survival data [32]. Heusner *et al* and Ludwig *et al* because they were only reporting on trans-arterial chemoperfusion [31, 33]. Pingpank *et al* conducted a Phase I study without reporting survival data [30]. Teal *et al* published a case report without actual survival data [34].

Then all articles were excluded that did not report a subset of uveal melanoma patients separately; Abbot *et al*, Magge *et al* and Hughes *et al* (the only phase III trial published on hepatic perfusion so far) did a combined report of patients with liver metastases from both uveal and cutaneous melanoma [37, 39, 40]. While Dewald *et al*, Rizell *et al*, Vogl *et al* and Libutti *et al* combined patients with liver metastases from uveal melanoma and patients with liver metastases from other cancers in their reports [35, 36, 38, 41].

Finally, all remaining articles were grouped with their research group or reporting hospital and compared to check for possible overlaps in reported patients by looking at years of inclusion. After this comparison only the articles with the highest number of patients reported from each research group or reporting hospital were included. This led to the exclusion of 14 articles, further specified in the four tables below:

Table S1. Studies from the Netherlands

1st Author	Year	Included	No. of Centres (City)	Number of Patients	Technique	Years of inclusion
Noter [43]	2004	No	1 (Leiden)	8	IHP	1999 - 2002
van Iersel [44]	2008	No	1 (Leiden)	13	IHP	1995 - 2006
van Iersel [45]	2014	No	1 (Leiden)	3	IHP	2007 - 2008
de Leede [63]	2016	Yes	2 (Leiden, Rotterdam)	31	IHP	1999 - 2009
Meijer [64, 65]	2019 + 2021	Yes	1 (Leiden)	35	PHP	2014 - 2017
van Etten [46]	2004	No	2 (Rotterdam)	1	IHP	?
Verhoef [47]	2008	No	2 (Rotterdam)	1	IHP	?
van Etten [48]	2009	No	2 (Rotterdam)	8	IHP	2002 - 2006

Table S2. Studies from Germany

1st Author	Year	Included	No. of Centres (City)	Number of Patients	Technique	Years of inclusion
Brüning [59]	2020	Yes	1 (Hamburg)	19	PHP	2014 - 2019
Dewald [60]	2021	Yes	1 (Hannover)	30	PHP	2014 - 2019
Kirstein [49]	2017	No	1 (Hannover)	11	PHP	2014 - 2016
Vogl [50]	2017	No	7 (Berlin, Frankfurt, Trier, Göttingen, Hamburg, Hannover, Heidelberg)	18	PHP	2012 - 2016
Schönfeld [51]	2020	No	1 (Hannover)	30	PHP	2014 - 2019
Artzner [52]	2019	No	1 (Tübingen)	16	PHP	2015 - 2018
Estler [61]	2021	Yes	1 (Tübingen)	29	PHP	2015 - 2020

Table S3. Studies from Sweden

1st Author	Year	Included	No. of Centres (City)	Number of Patients	Technique	Years of inclusion
Olofsson [53]	2014	No	1 (Göteborg)	34	IHP	2005 - 2011
Ben-Shabat [58]	2016	Yes	1 (Göteborg)	68	IHP	1989 - 2013

Table S4. Studies from the United States of America and the United Kingdom

1st Author	Year	Included	No. of Centres (City)	Number of Patients	Technique	Years of inclusion
Forster [54]	2014	No	1 (Tampa, FL)	5	PHP	2008 - 2013
Karydis [62]	2018	Yes	2 (Tampa, FL & Southampton, UK)	51	PHP	2008 - 2016
Alexander [55]	2000	No	1 (Bethesda, MD)	22	IHP	1994 - 1999
Alexander [57]	2003	Yes	1 (Bethesda, MD)	29	IHP	1997 - 2002
Varghese [56]	2010	No	1 (Bethesda, MD)	17	IHP	?

FL: Florida, UK: United Kingdom, MD: Maryland
